# Metagenomic and metatranscriptomic profiling of bronchoalveolar lavage fluid identifies microbial and host biomarkers of drug-resistant tuberculosis

**DOI:** 10.3389/fcimb.2025.1726935

**Published:** 2026-01-29

**Authors:** Haiqing Zhang, Limao Zhang, Bin Yang, Chunjing Gao, Huimei Liu, Yunshi Zhang, Xiaoyou Chen

**Affiliations:** 1 Department of Tuberculosis, Xuzhou Hospital, Beijing Ditan Hospital Affiliated to Capital Medical University, Xuzhou, Jiangsu, China; 2 Center for Infectious Diseases, Vision Medicals Co., Ltd, Guangzhou, Guangdong, China; 3 Department of Science and Education, Xuzhou Hospital, Beijing Ditan Hospital Affiliated to Capital Medical University, Xuzhou, Jiangsu, China; 4 Xuzhou Hospital, Beijing Ditan Hospital Affiliated to Capital Medical University, Xuzhou, Jiangsu, China

**Keywords:** drug resistance, host response, microbiome, *Mycobacterium tuberculosis*, transcriptomics

## Abstract

**Background:**

Drug-resistant tuberculosis (DR-TB) undermines global TB control, yet how resistant *Mycobacterium tuberculosis* strains interact with the lung microbiome, phage communities, and local host immunity remains poorly defined.

**Methods:**

In a prospective cohort of 130 pulmonary TB patients (49 DR-TB, 81 drug-susceptible TB [DS-TB] patients), bronchoalveolar lavage fluid (BALF) was subjected to paired metagenomic and transcriptomic profiling. Microbial and bacteriophage community structures were assessed by diversity metrics and differential abundance testing, whereas host responses were characterized by gene expression, pathway enrichment, and immune cell deconvolution. A Random Forest model was trained to evaluate the diagnostic potential of host transcriptional signatures.

**Results:**

DR-TB airways presented distinct microbial beta diversity, with enrichment of Streptococcus spp. and streptococcal-targeting phages (e.g., Javan variants, phi-Ssu5SJ28rum). Transcriptomic analysis revealed 494 differentially expressed genes, which were associated with increased oxidative phosphorylation, suppressed ion channel and transporter activity, and enrichment of extracellular matrix remodeling pathways. Immune profiling demonstrated a significant reduction in γδ T cells in DR-TB patients (P = 0.0059). An 8-gene host-derived signature (*ARHGEF5, PTGES3L, GAL3ST1, RANBP17, ACTA2_AS1, CBY3, MAMSTR, and LOC102031319*) discriminated DR-TB from DS-TB with high accuracy (AUC = 0.837).

**Conclusion:**

This dual-omics study defines the airway niche of DR-TB as a convergence of microbial dysbiosis, phage imbalance, and host immune–metabolic dysfunction. By uncovering DR-TB–specific microbial and transcriptional signatures, and deriving a predictive host-based classifier, our findings provide mechanistic insights and highlight novel opportunities for microbiome- and host-directed interventions in drug-resistant tuberculosis.

## 1 Introduction

Tuberculosis (TB), caused by *Mycobacterium tuberculosis* (Mtb), remains a leading cause of morbidity and mortality worldwide. The rise of drug-resistant TB (DR-TB) poses a critical threat to global TB control, undermining treatment efficacy and worsening clinical outcomes. While adaptive immunity in TB has been extensively studied, early host–pathogen interactions at the respiratory mucosal surface and their modulation by the local microbiome are increasingly recognized as key determinants of infection dynamics (Lange et al., 2019; Walker et al., 2022). Antibiotic therapy itself induces profound and persistent dysbiosis, particularly in barrier sites such as the lung, which may compromise immune homeostasis and increase susceptibility to reinfection.

The lower respiratory tract (LRT), once considered sterile, is now known to harbor a low-biomass but immunologically relevant microbial community (bacteriome, virome) distinct from that of the upper airway (Hong et al., 2016). These commensals dynamically interact with airway epithelial cells (AECs) and local immune populations, including γδ T cells, NK cells, and MAIT cells, thereby shaping the early containment of Mtb and bridging to adaptive immunity (Gupta et al., 2018; Howard and Khader, 2020). Importantly, drug-resistance–conferring mutations in Mtb (e.g., rpoB mutations) alter cell wall lipid composition and bacterial physiology, potentially influencing interactions with both the microbiome and host immune pathways. Emerging evidence links TB susceptibility and progression to perturbations in the gut–lung axis and the pulmonary microbiome (Gupta et al., 2018). Indeed, recent studies have demonstrated that integrating host transcriptional profiling with respiratory metagenomics can significantly increase the diagnostic accuracy for lower respiratory tract infections (Mick et al., 2023; Zou et al., 2025b). However, a critical knowledge gap persists: the interplay between drug-resistant Mtb strains, the lung microbiome-including bacteriophage and local immune-metabolic responses at the airway interface remains largely unexplored (Lin et al., 2021; Valdez-Palomares et al., 2021; Fonseca et al., 2025).

To address this gap, we systematically collected bronchoalveolar lavage fluid (BALF) from a prospective cohort of pulmonary TB patients and applied a dual-omics strategy integrating metagenomic profiling of microbial and phage communities with host transcriptomic analysis. This approach enabled us to identify DR-TB–specific microbial and phage signatures, identify host immune–metabolic pathways associated with resistance, and evaluate the diagnostic potential of host transcriptional biomarkers. By directly interrogating the distal airway niche, our study provides novel insights into the ecological and immunological basis of DR-TB pathogenesis and highlights potential avenues for host- and microbiome-directed interventions.

## 2 Materials and methods

### 2.1 Study design, patient cohort, and ethics statement

**Figure 1:**
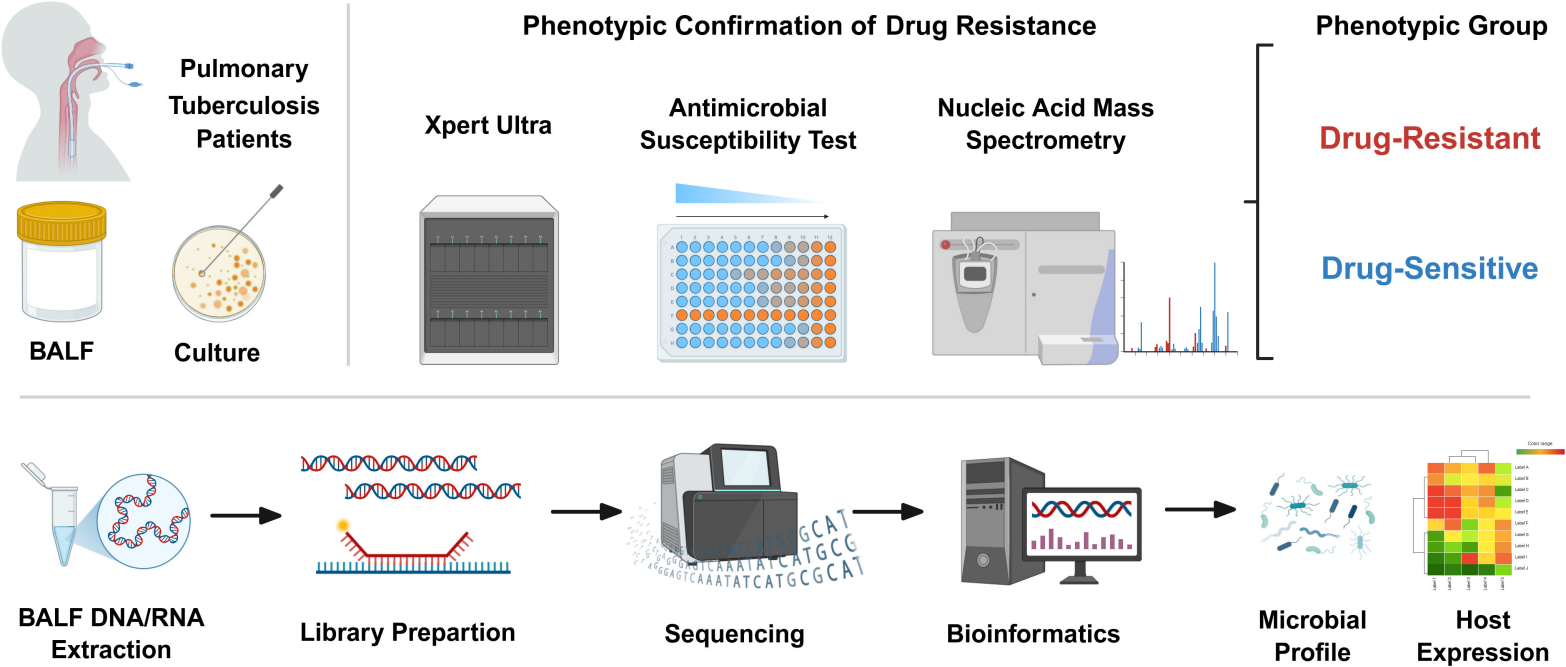
Study design integrating phenotypic drug resistance confirmation with dual-omics profiling of BALF. Flowchart depicting study procedure: (upper panel) Pulmonary tuberculosis patients were stratified into drug-resistant TB (DR-TB) or drug-sensitive TB (DS-TB) groups through a triangulated phenotyping strategy that combined culture-based antimicrobial susceptibility testing, Xpert MTB/RIF Ultra, and nucleic acid mass spectrometry. According to WHO criteria, isolates resistant to ≥1 first- or second-line drug were classified as DR-TB, whereas isolates susceptible to all tested drugs were defined as DS-TB. (lower panel) From the same bronchoalveolar lavage fluid (BALF) specimens, paired DNA and RNA were extracted for metagenomic and metatranscriptomic library preparation. High-throughput sequencing and standardized bioinformatics pipelines enabled integrative characterization of the airway microbiome (including bacteriophages) and host transcriptional responses within the same anatomical niche.

From December 2024 to May 2025, we enrolled 130 patients with confirmed pulmonary tuberculosis (TB) in a prospective observational cohort in Jiangsu Province. A definitive diagnosis required bacteriological or molecular confirmation (**Figure 1**): (1) Acid-fast bacilli (AFB) smear positivity in sputum, bronchoalveolar lavage fluid (BALF), or pleural fluid; (2) positive culture of Mtb complex; or (3) molecular testing (e.g., GeneXpert MTB/RIF) detecting Mtb DNA. Crucially, to ensure accurate stratification into DR-TB and drug-sensitive TB (DS-TB) groups, only patients with definitive phenotypic drug susceptibility testing (DST) results or molecular confirmation of drug resistance were included in the final cohort. Patients diagnosed solely on clinical grounds without microbiological evidence of drug susceptibility profiles were excluded from this study.

Confirmed Mtb isolates were subjected to DST via commercial microplate dilution kits in 7H9 broth, and their sensitivities to 11 anti-TB drugs (ATDs) at both low and high concentrations were tested. Kits were provided by Autobio Diagnostics (2018–2019) and Encode Medical Engineering (2020 onward). Testing followed the manufacturer’s protocols and CLSI guidelines. The inoculum size was standardized at 200 µL/well. Cultures were checked for contamination after 7 days, with final readings at 12–13 days if uncontaminated. The drugs tested included four first-line agents-isoniazid (0.20, 0.80 μg/ml), rifampicin (4.0, 8.0 μg/ml), streptomycin (4.0, 8.0 μg/ml), and ethambutol (2.5, 5.0 μg/ml)-and seven second-line agents: levofloxacin (2.0, 8.0 μg/ml), moxifloxacin (0.5, 2.0 μg/ml), para-aminosalicylic acid (PAS; 2.0, 8.0 μg/ml; system updated Oct 2019), rifabutin (0.75, 3.0 μg/ml), amikacin (1.0, 4.0 μg/ml), kanamycin (2.5, 10.0 μg/ml), and capreomycin (2.5, 10.0 μg/ml). The susceptible H37Rv strain (ATCC 27294) served as a control, with interlaboratory testing for quality assurance.

The GeneXpert MTB/RIF assay detected Mtb and rifampicin resistance: samples were mixed 1:2 with Xpert reagent, incubated at room temperature for 15 min, and processed on the GeneXpert system. Failed or indeterminate samples were retested or excluded.

For PCR/MALDI-TOF MS, nucleic acid extraction was performed under BSL-2/3 conditions. Colonies from solid or liquid cultures (0.5 McFarland) were suspended in PrepMan Ultra, vortexed, heat-killed (90-100°C, 30 min), centrifuged, and supernatants stored. Extracts were diluted 1:10 before testing. A multiplex PCR amplified 24 target regions in 10 genes, followed by cleanup and dephosphorylation. A second multiplex PCR enabled single-base extension with detection probes. Extension products were resin-cleaned (Chip Prep Module) and spotted onto matrix-loaded chips for MALDI-TOF MS analysis (MassARRAY system).

Patients were stratified into drug-resistant TB (DR-TB; n = 49, resistant to ≥1 first- or second-line drug) and drug-sensitive TB (DS-TB; n = 81, pan-susceptible) exclusively based on rigorous phenotypic classification. All data originated from archived clinical materials at Xuzhou Infectious Disease Hospital under a no-contact protocol approved by the Institutional Review Board (IRB Approval No.: XCYPJ-2024112603-01). Written informed consent was obtained for sampling and secondary research use of specimens.

### 2.2 DNA/RNA extraction, library construction, and sequencing

For metagenomic DNA sequencing (mNGS-DNA), 1 mL of BALF was subjected to host nucleic acid depletion with 1 U of benzonase (Sigma) and 0.5% Tween-20 (Sigma) and incubated at 37°C for 5 min. Six hundred microliters of the mixture was transferred to tubes containing 500 µL of ceramic beads for bead beating (Minilys Homogener, Tiangen, China). Nucleic acids from 400 µL pretreated samples were extracted via a QIAamp UCP Pathogen Mini Kit (Qiagen, Germany) and eluted in 60 µL of buffer. For DNA quantification, a Qubit dsDNA high sensitivity assay (Invitrogen, USA) was used.

For metatranscriptomic RNA sequencing (mNGS-RNA), 1 mL of BALF was centrifuged at 12,000 rpm for 10 min. Pellets were lysed in TRIzol LS (Thermo Fisher Scientific, USA), followed by RNA extraction via a Direct-zol RNA Miniprep Kit (Zymo Research, USA).

Libraries were prepared from 30 µL of DNA via the Nextera DNA Flex Kit (Illumina, USA) and from 10 µL of RNA via the Ovation Trio RNA-Seq Kit (NuGEN, USA). Library concentrations were measured via the Qubit dsDNA HS assay, and quality was assessed via an Agilent 2100 Bioanalyzer with a high-sensitivity DNA kit. Sequencing was performed on an MGI G99 platform (single-end, 75 bp). The raw sequence data reported in this paper have been deposited in the Genome Sequence Archive (Genomics, Proteomics & Bioinformatics 2025) (Zhang et al., 2025) in National Genomics Data Center (Nucleic Acids Res 2025), China National Center for Bioinformation/Beijing Institute of Genomics, Chinese Academy of Sciences (GSA: CRA034880) that are publicly accessible at https://ngdc.cncb.ac.cn/gsa.

### 2.3 Microbial annotation, community structure comparison, and differential taxon analysis

Microbial composition analysis followed a validated mNGS pipeline (Diao et al., 2023; Han et al., 2025; Lin et al., 2025; Sun et al., 2025; You et al., 2025): Briefly, Trimmomatic was used to remove low-quality and duplicate reads as well as adapter sequences (Bolger et al., 2014); Reads were mapped to the human reference genome (hg38) using SNAP v1.0beta to exclude host-derived sequences (Miller et al., 2019); and ribosomal RNA sequences were filtered with SortMeRNA v4.3.7. Taxonomic profiles were generated using Kraken2 v2.0.7 and Bracken v2.5 with default reference databases (https://benlangmead.github.io/aws-indexes/k2) (Diao et al., 2023; Ho et al., 2023). To correct for sequencing depth, microbial reads were normalized as reads per million (RPM). Potential contaminants were identified by comparing taxon abundance between bronchoscope background controls and negative controls (PBS or sterile water). Taxa with median relative abundance >50% higher in background controls or with mean relative abundance >0.1% in negative controls were excluded as contaminants.

Phage annotation was performed by BLAST alignment (word size = 18, e-value = 0.0005, culling limit = 1) against a curated phage database (CPD) comprising 26,159 representative genomes (Haddock et al., 2023). Phage contig quality was further assessed, and microbial/phage abundances were expressed as relative abundances (Haddock et al., 2023).

α-diversity indices (Shannon, Simpson, Chao1, ACE) for both DNA- and RNA-based profiles were calculated using the vegan R package (Ren et al., 2021). β-diversity was assessed using Bray-Curtis dissimilarity and compared between groups by PERMANOVA (adonis, 999 permutations, vegan package). Principal coordinates analysis (PCoA) was used for visualization of community structure. Differentially abundant taxa and bacteriophages were identified using linear discriminant analysis effect size (LEfSe), with LDA score > 2 and FDR-adjusted p < 0.05 considered significant. HUMAnN2 software (Truong et al., 2015; Beghini et al., 2021), which based on metagenomic data, was applied for analyzing the abundance of microbial pathways, and then displayed by Kyoto Encyclopedia of Genes and Genomes (KEGG) (Caspi et al., 2020).

### 2.4 Gene expression, transposable element expression, and cell-type composition analysis

Host transcriptome data were aligned to the human reference genome (hg38) using HISAT2 with default settings, and gene-level quantification was performed with FeatureCounts (Subread package v2.0.0 (http://subread.sourceforge.net/)) (Liao et al., 2014; Kim et al., 2019). Trimmed reads were mapped using STAR (predefined parameters). Normalization and batch effect correction were applied prior to differential expression analysis with DESeq2 (Benjamini–Hochberg FDR ≤ 0.05; |log_2_ fold change| ≥ 1) (Love et al., 2014). Differentially expressed genes (DEGs) were subjected to gene set enrichment analysis (GSEA) via the REACTOME, Kyoto Encyclopedia of Genes and Genomes (KEGG), and Gene Ontology (GO) databases (Subramanian et al., 2005; Kanehisa et al., 2017; Gillespie et al., 2022). Cell-type composition was inferred from bulk transcriptomes using CIBERSORT with the LM22 signature matrix (1,000 permutations) (Steen et al., 2020). Nonparametric comparisons of continuous variables (latent variables and cell fractions) between groups were performed with the Mann-Whitney U test, and multiple testing was corrected by the Benjamini-Hochberg method (adjusted p < 0.05 considered significant).

### 2.5 Model construction for drug-resistant tuberculosis classification

An ensemble machine learning framework was developed to distinguish drug-resistant TB (DR-TB) from drug-sensitive TB (DS-TB) using BALF RNA sequencing-derived host transcriptomic signatures. Differential expression was first assessed using DESeq2 (median-of-ratios normalization; variance-stabilizing transformation) (Segata et al., 2011), and genes with p < 0.05 were retained as candidate biomarkers; batch effects were corrected using ComBat-seq. To account for possible confounders, we conducted multivariate regression analysis using clinical data of patients (**Supplementary Table S1**) as covariates. Genes with a significance level of p<0.05 in this adjusted model were retained for constructing the classifier. Subsequently, a Random Forest (RF) classifier (ranger package) was then trained after sequential forward feature selection with 1,000 bootstrap iterations, and to address class imbalance, the majority class was undersampled in each iteration. Hyperparameters were optimized by nested stratified 10-fold cross-validation with mtry (candidate features per split) tuned across [√n, n/3], ntree (number of trees) varied from 500-2,000, and minimal node size determined by minimizing Gini impurity. Model performance was evaluated on an independent 30% hold-out set and reported as area under the ROC curve (AUC), sensitivity, specificity, and F1-score, with 95% confidence intervals estimated via bootstrapping, and calibration was assessed by the Brier score. Model interpretability was achieved using SHapley Additive exPlanations (SHAP; shap Python library v0.41.0 via reticulate), where global feature importance was summarized as mean absolute SHAP values, sample-level contributions were visualized with force plots, and dependence plots illustrated the relationship between gene expression values (color-coded, high = yellow and low = purple) and their contribution to predictions. All analyses were performed in R v4.1.0 with DESeq2 v1.32.0, mlr3 v0.13.0, ranger v0.13.1, and reticulate v1.22.

### 2.6 Contamination control

Negative controls (PBS or sterile water) were processed in parallel with BALF specimens. In addition, sterile cotton swabs moistened with sterile deionized water were used to wipe the surfaces of the centrifuge and biosafety cabinet, generating a background microorganism profile for our laboratory. Potential contaminant taxa were identified by prevalence filtering (decontam v1.20.0) and excluded.

## 3 Results

### 3.1 Microbial composition and diversity of DR-TB vs. DS-TB

We profiled 130 patients (**Supplementary Table S1**, **Supplementary Table S2**) with active pulmonary tuberculosis (TB) confirmed by both clinical and microbiological criteria. Based on drug susceptibility testing (DST), the cohort included 81 cases of drug-susceptible TB (DS-TB) and 49 cases of drug-resistant TB (DR-TB). Within the DR-TB group, 33 patients had isoniazid monoresistance (INH-MR), 12 had rifampicin monoresistance (RFP-MR), 26 met criteria for multidrug-resistant TB (MDR-TB), and 39 were classified as polyresistant TB (PR-TB).

**Figure 2:**
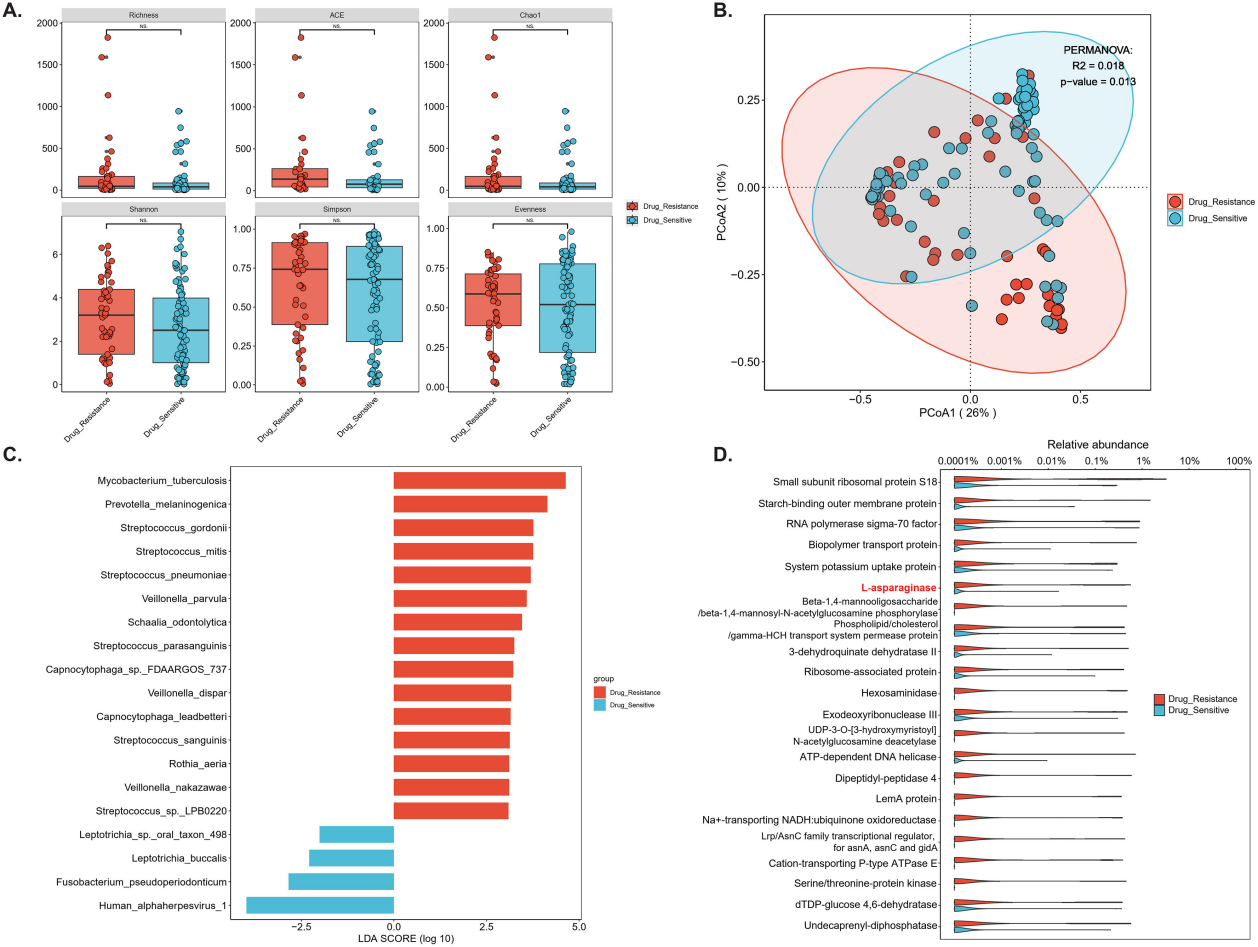
Comparative microbiome profiles in drug-resistant versus drug-sensitive TB. **(A)** Alpha diversity metrics (Shannon index, Simpson index, richness, evenness, Chao1 and ACE) comparing drug-resistant (red) and drug-sensitive (blue) groups. **(B)** Beta diversity analysis based on Bray–Curtis dissimilarity, visualized by principal coordinates analysis (PCoA). Group separation was statistically supported by PERMANOVA (R^2^ = 0.018, p = 0.013). **(C)** LEfSe identifying discriminative taxa enriched in either group. Only taxa with LDA score > 2 and p-value < 0.05 are displayed. **(D)** Differentially abundances microbial functional pathways. Features highlighted in red demonstrate denote functions significantly enriched in drug-resistant samples, several of which are associated with Mycobacterium tuberculosis metabolic adaptation and host interaction. *p < 0.05; **p < 0.01; ***p < 0.001; N.S., not significant.

Metagenomic DNA data generated a median of 16.7 million clean reads (IQR 13.6-20.5 million) per patient, with microbial reads accounting for 10.55% (IQR 4.45-33.72%, **Supplementary Table S3**) after host filtering. Metagenomic profiling of BALF microbiota, including bacteria, eukaryotes, and viruses, revealed a trend toward higher alpha diversity in DR-TB compared with DS-TB, although the difference did not reach statistical significance (**Figure 2A**; p > 0.05). In contrast, beta diversity analysis based on Bray-Curtis dissimilarity demonstrated significant compositional separation between groups (**Figure 2B**; PERMANOVA, p < 0.05). LEfSe identified taxa discriminating the two phenotypes. As expected, Mtb was significantly enriched in DR-TB samples (log10 LDA score = 4.63, p = 0.017; **Figure 2C**), accompanied by increased abundance of *Prevotella melaninogenica* and *Streptococcus* spp. In contrast, Human alphaherpesvirus 1, *Fusobacterium pseudoperiodonticum*, and *Leptotrichia* spp. were more abundant in DS-TB samples (**Figure 2C**, **Supplementary Figures S1A, B**). Correlation analysis further revealed a broadly negative association between Mtb abundance and other commensal taxa (**Supplementary Figure S1C**; p < 0.05), suggesting competitive exclusion or ecological disruption in the DR-TB airway niche.

Functional profiling of metabolic pathways demonstrated distinct differences between DR-TB and DS-TB isolates (**Figure 2D**). DR-TB strains exhibited significantly higher abundance of enzymes critical for intracellular survival and virulence, including L-asparaginase (asparagine utilization), a starch-binding outer membrane protein (nutrient uptake and membrane integrity), and UDP-3-O-[3-hydroxymyristoyl] N-acetylglucosamine deacetylase (lipid A biosynthesis). Conversely, DS-TB strains were enriched in enzymes implicated in host-pathogen interactions and cell wall remodeling, such as hexosaminidase and dipeptidyl-peptidase 4 (**Figure 2D**).

### 3.2 Bacteriophage composition and diversity of DR-TB vs. DS-TB

**Figure 3:**
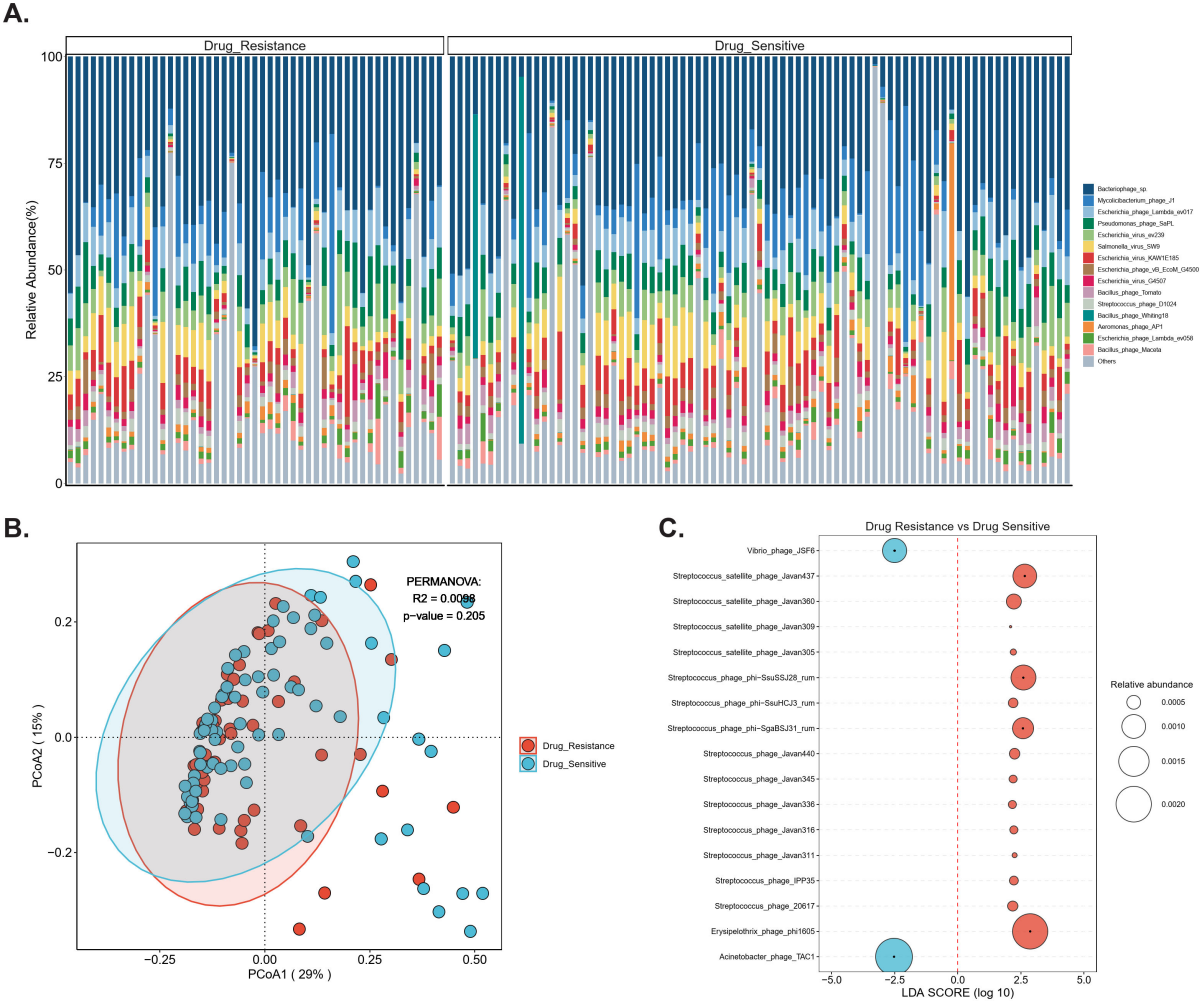
Comparative bacteriophage profiles in drug-resistant versus drug-sensitive TB. Comparative analysis of bacteriophages features between drug-resistant and drug-sensitive isolates. **(A)** Bar plot display the proportional distribution of major phage taxa across individual BALF samples. **(B)** Beta diversity (PCoA) between drug resistant (red) and drug sensitive (blue) (PERMANOVA: R^2^ = 0.0098, p = 0.205). **(C)** LEfSe highlighting phage taxa enriched in either group. Only taxa with LDA score > 2 and p-value < 0.05 are shown. Circle size denotes relative abundance.

Bacteriophage profiling revealed Bacteriophage sp. as the dominant taxon across all samples, followed by *Mycolicibacterium* phage J1 and Escherichia phage Lambda evo17 (**Figure 3A**). Hierarchical clustering based on relative abundance demonstrated distinct community patterns segregating DR-TB (blue) and DS-TB (red) groups. DR-TB samples were enriched in multiple streptococcal-associated phages, including *Streptococcus satellite* phage variants (e.g., phi-Ssu5SJ28rum, Javan440, IPP35) and *Erysipelothrix* phage phi1805, whereas DS-TB samples exhibited higher abundance of Vibrio phage JSF6 and Acinetobacter phage TAC1 (**Figure 3A**). Principal coordinates analysis (PCoA) of Bray-Curtis dissimilarity suggested partial separation along PCoA1 (29% variance) and PCoA2 (15% variance), although PERMANOVA did not reach statistical significance (R² = 0.0098, p = 0.205; **Figure 3B**). Notably, alpha diversity was significantly higher in DR-TB compared with DS-TB, as indicated by increased ACE and Chao1 indices (p < 0.05; **Supplementary Figure S2**).

LEfSe analysis further confirmed discriminative phage taxa between groups (**Figure 3C**). DR-TB samples were characterized by enrichment of diverse *Streptococcus* phages (e.g., phi-Ssu5SJ28rum, phi-SsuHCJ3rum, Javan440, Javan345, Javan336, and phage 20617), satellite phages (Javan437, Javan360, Javan309, Javan305), and *Erysipelothrix* phage phi1805, all with high positive log10 LDA scores. In contrast, Vibrio phage JSF6 and *Acinetobacter* phage TAC1 were preferentially enriched in DS-TB samples (**Figure 3C**). These distinct phage community signatures indicate phenotype-specific airway viromes and suggest potential contributions to microbiome dysbiosis, horizontal gene transfer, or modulation of bacterial fitness in the DR-TB niche (**Figures 3A-C**).

### 3.3 Host transcription profiles: identification of differentially expressed genes

**Figure 4:**
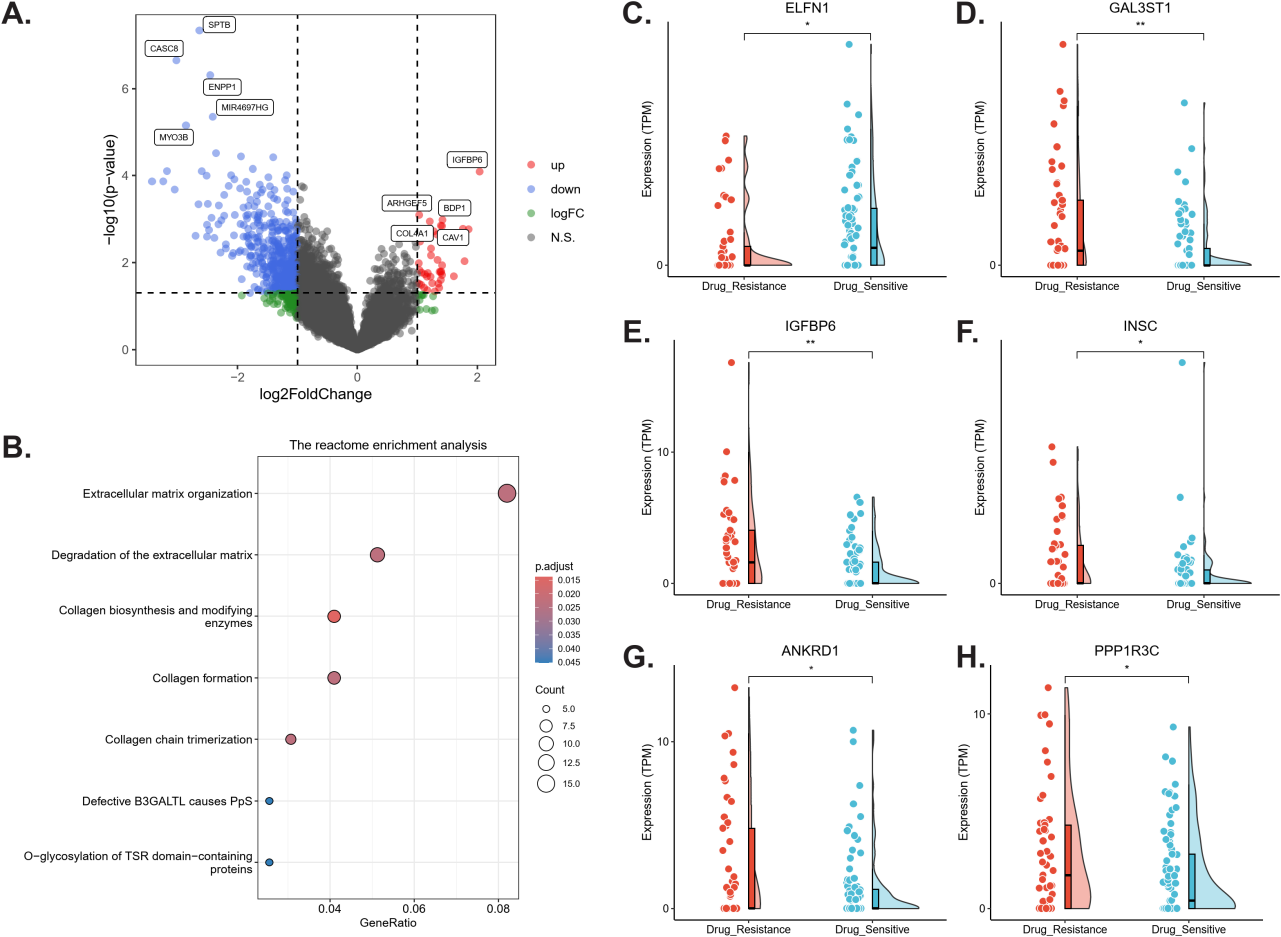
Differential host transcriptomic profiles in drug-resistant versus drug-sensitive TB. Comparative transcriptomic analysis between drug-resistant (red) and drug-sensitive (blue) conditions. **(A)** Volcano plot of differentially expressed genes (DEGs). Genes with |log_2_ fold change| ≥1 and adjusted p-value < 0.05 were considered significant. **(B)** Reactome pathway enrichment analysis of DEGs, highlighting pathways related to extracellular matrix remodeling and collagen biosynthesis. Pathways with adjusted p-value < 0.05 were retained. **(C–H)** Expression distributions of representative DEGs. ELFN1 was downregulated in drug-resistant TB, while GAL3ST1, IGFBP6, INSC, ANKRD1, and PPP1R3C were upregulated. These genes formed part of the diagnostic signature identified in downstream machine-learning analyses. *p < 0.05; **p < 0.01; N.S., not significant.

RNA sequencing (**Supplementary Table S4**) identified 494 DEGs between DR-TB and DS-TB samples (|log_2_FC| ≥ 1, p < 0.05), including 39 upregulated and 455 downregulated in DR-TB (**Figure 4A**). Representative genes are shown in **Figures 4C-H**, where *ARHGEF5 (Rho Guanine Nucleotide Exchange Factor 5)*, IGFBP6 (*insulin like growth factor binding protein 6), PTGES3L (Prostaglandin E Synthase 3-Like)*, and *BDP1 (BDP1 general transcription factor IIIB subunit)* were significantly upregulated, whereas ENPP1 (*ectonucleotide pyrophosphatase/phosphodiesterase 1)*, *RANBP17 (RAN Binding Protein 17)* and so on were downregulated in DR-TB relative to DS-TB (all p < 0.05). Pathway analyses revealed coherent transcriptional reprogramming: Reactome analysis indicated enrichment of collagen biosynthesis and extracellular matrix remodeling pathways (adjusted p < 0.05; **Figure 4B**); GO analysis showed downregulation of processes linked to transmembrane transport and ion channel activity (adjusted p < 0.05; **Supplementary Figure S2A**); and KEGG analysis highlighted significant alterations in “Neuroactive ligand-receptor interaction” (adjusted p < 0.01) and the “Hippo signaling pathway” (adjusted p < 0.05; **Supplementary Figure S2B**). GSEA further demonstrated upregulation of oxidative phosphorylation (OxPhos)-related pathways-including ATP synthesis–coupled electron transport, respiratory chain complex assembly, and mitochondrial respirasome function (adjusted p < 0.05; **Supplementary Figure S2C**)-indicating enhanced mitochondrial energy metabolism in DR-TB, accompanied by suppressed signaling related to ion transporters and channel activity.

**Figure 5:**
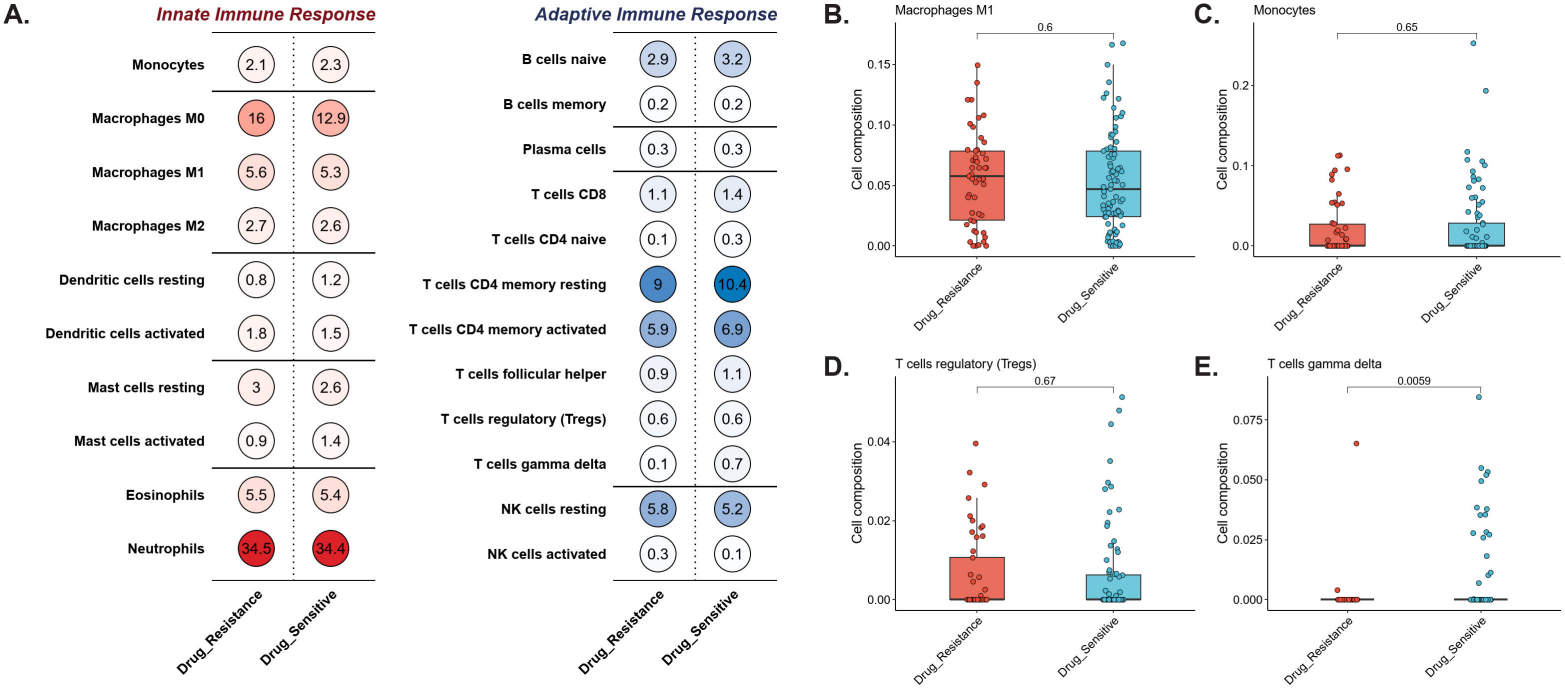
Comparative immune cell composition in drug-resistant vs. versus drug-sensitive conditions. **(A)** Relative proportions of innate and adaptive immune cell subsets inferred from bulk transcriptomes by CIBERSORT. Circle size denotes the estimated fraction of each cell type, and color intensity reflects mean abundance within each group. **(B–E)** Distribution of selected immune cell subsets showing group-level differences: macrophages M1 **(B)**, monocytes **(C)**, regulatory T cells (Tregs) **(D)**, and γδ T cells **(E)**. Drug-resistant samples are denoted in red and drug-sensitive samples in blue. Among these subsets, γδ T cells were significantly reduced in the drug-resistant group (p = 0.0059), whereas other populations showed no significant differences.

Computational immune deconvolution (CIBERSORT) of BALF transcriptomes revealed broadly comparable immune cell composition between DR-TB and DS-TB patients (**Figure 5A**). Neutrophils constituted the largest fraction (~34.5% DR-TB vs. ~34.4% DS-TB), followed by M0 macrophages (16.0% vs. 12.9%), CD4^+^ resting memory T cells (9.0% vs. 10.4%), M1 macrophages (~5.6% vs. ~5.3%), and activated CD4^+^ memory T cells (~5.9% vs. ~6.9%). No significant differences were observed in monocytes, M0 macrophages, or regulatory T cells (p > 0.05; **Figures 5B–D**). In contrast, γδ T cells were markedly reduced in DR-TB compared with DS-TB (0.14% vs. 0.74%, p = 0.0059; **Figure 5E**), highlighting a selective deficiency in this innate-like T cell subset that may contribute to impaired early immune containment of drug-resistant infection.

### 3.4 Diagnostic model for DR-TB discrimination

**Figure 6:**
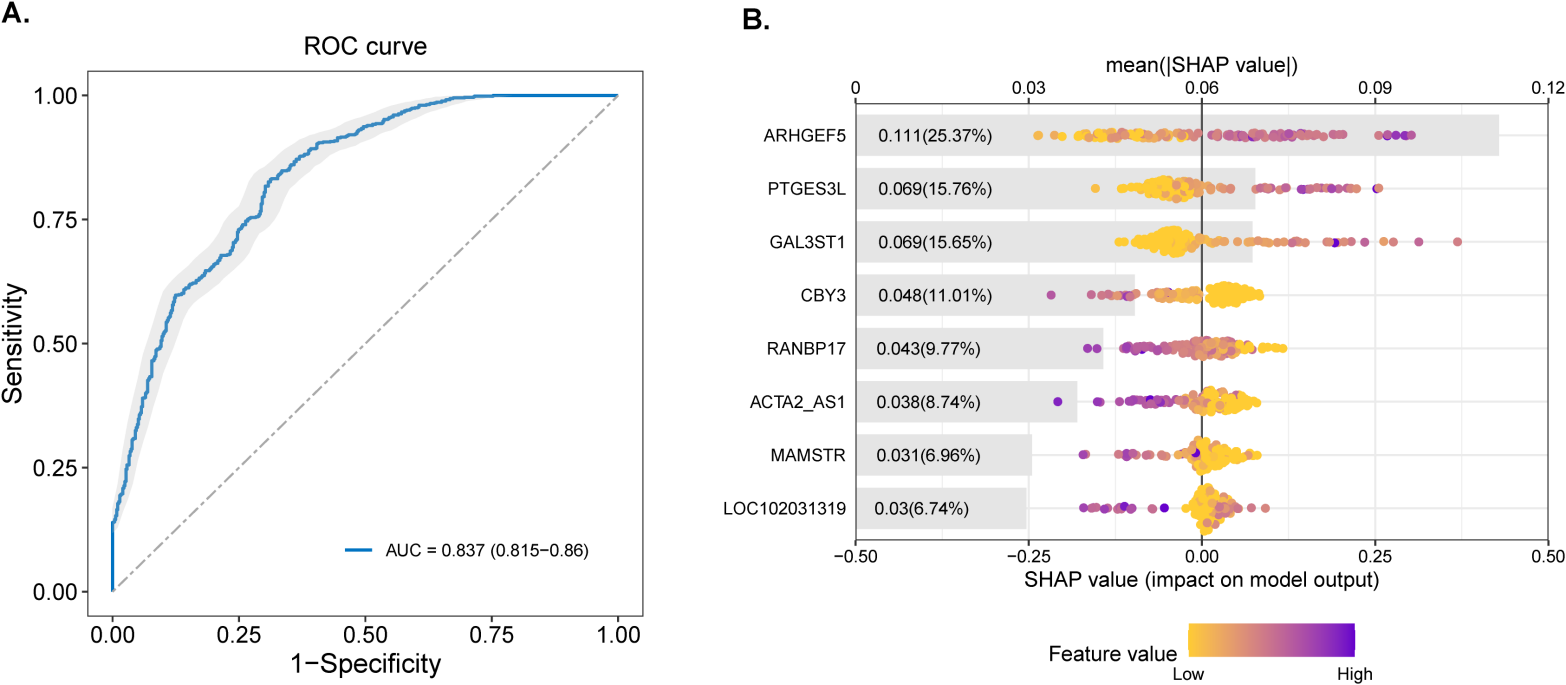
Diagnostic performance and feature importance of predictive model. **(A)** ROC curve of the Random Forest classifier distinguishing drug-resistant (DR-TB) from drug-sensitive TB (DS-TB) using the eight-gene transcriptional signature. The model achieved an AUC of 0.837 (95% CI: 0.815–0.86) under stratified cross-validation. Shaded area indicates the 95% confidence interval. **(B)** Feature importance ranked by SHAP values. Gray bars represent the average contribution of each gene, and scattered points indicate individual sample-level SHAP values, with color denoting feature expression (yellow: high, purple: low). *ARHGEF5, PTGES3L, GAL3ST1*, and *RANBP17* contributed most strongly to model discrimination, consistent with their differential expression between groups.

To evaluate the diagnostic potential of host transcriptional signatures, we constructed a Random Forest classifier based on BALF-derived DEGs. An optimal 8-gene panel including *ARHGEF5*, *PTGES3L, GAL3ST1 (*Galactose-3-O-Sulfotransferase 1*), RANBP17 (*RAN Binding Protein 17*), ACTA2_AS1 (*ACTA2 Antisense RNA 1*), CBY3 (*Chibby Family Member 3*), MAMSTR* (MEF2 Activating Motif and SAP Domain Containing Transcriptional Regulator), and *LOC102031319* (Uncharacterized Locus 102031319)) discriminated DR-TB from DS-TB with high accuracy, achieving an AUC of 0.837 (95% CI: 0.815-0.86; **Figure 6A**). Model interpretability analysis using SHAP values ranked gene contributions to classification (**Figure 6B**). *ARHGEF5* exerted the strongest influence (mean |SHAP| = 0.111; 25.37% contribution), followed by *PTGES3L* (0.069; 15.76%) and *GAL3ST1* (0.069; 15.65%). Notably, higher expression of *ARHGEF5, PTGES3L and GAL3ST1* (purple points) was consistently associated with accurate DR-TB prediction, whereas lower expression (yellow points) weakened predictive performance.

## 4 Discussion

To the best of our knowledge, this study represents the first comprehensive dual-omics analysis of BALF from patients with DR-TB and DS-TB. By simultaneously characterizing the lung microbiome-including bacteria, eukaryotes, viruses, and bacteriophages-via metagenomics, and the host immune-metabolic response via transcriptomics within the same anatomical compartment, we uncovered previously unrecognized pathogen-host dynamics in DR-TB. The principal innovations of this work include: (i) identification of DR-TB-specific microbial signatures, notably enrichment of Streptococcus species and streptococcal-targeting phages (e.g., Javan variants, phi-Ssu5SJ28rum), implicating antibiotic-driven dysbiosis and phage-mediated interactions in DR-TB pathogenesis; (ii) demonstration that host transcriptional profiles in BALF can discriminate DR-TB from DS-TB, marked by upregulated oxidative phosphorylation (OxPhos), suppression of ion channel and transporter activity, and depletion of γδ T cells; and (iii) development of an 8-gene BALF-based diagnostic classifier (including *ARHGEF5, PTGES3L, GAL3ST1, RANBP17, ACTA2_AS1*), which achieved an AUC of 0.837, highlighting the utility of host-derived biomarkers beyond conventional bacterial genotyping approaches. Together, these findings establish the distal airway as a critical compartment for DR-TB pathobiology and suggest new opportunities for host- and microbiome-directed interventions.

The microbial ecology of DR-TB displayed distinct adaptations relative to DS-TB. While alpha diversity did not differ significantly between groups, beta diversity was clearly distinct, consistent with niche specialization of resistant Mtb strains (Valdez-Palomares et al., 2021; Du et al., 2024). DR-TB samples exhibited consistent enrichment of Streptococcus species, a genus previously linked to TB treatment failure and recurrence (Wu et al., 2013; Shahzad et al., 2024). Enrichment of Streptococcus has also been reported in other respiratory conditions, such as community-acquired pneumonia (Yang et al., 2024b) and influenza infection (Zou et al., 2025a), where it often correlates with acute inflammation or secondary bacterial coinfection. However, unlike these acute settings, the expansion of Streptococcus in our DR-TB cohort likely reflects antibiotic-induced dysbiosis under chronic treatment pressure. This specific niche may not only represent a marker of drug resistance but could also facilitate *M. tuberculosis* persistence through metabolic cross-feeding or biofilm formation, distinct from the immune-stimulatory role observed in acute viral-bacterial coinfections (Zou et al., 2025a). This enrichment creates a permissive environment for streptococcal expansion and potentially facilitating Mtb persistence via interbacterial interactions. Importantly, phage communities were skewed toward Streptococcus-targeting phages in DR-TB, whereas DS-TB samples harbored more diverse phages such as Vibrio phage JSF6 and Acinetobacter phage TAC1. Given the role of phages in horizontal gene transfer and bacterial fitness modulation, such imbalances may reinforce the ecological advantages of resistant strains. Recent studies underscore the therapeutic potential of phages in mycobacterial infections, with engineered or humanized phages reducing bacterial burden in animal models (Yang et al., 2024a) and compassionate use successfully treating M. abscessus infections in humans (Azimi et al., 2019; Dedrick et al., 2019). Our findings extend this paradigm by linking phage composition to DR-TB specifically, suggesting the airway phageome as both a biomarker and a therapeutic target.

Host transcriptional responses revealed profound rewiring of metabolic and immune pathways in DR-TB. Upregulation of OxPhos-related genes indicates enhanced mitochondrial activity, in contrast to the glycolytic reprogramming typically adopted by macrophages during early Mtb infection to fuel antimicrobial responses (Cook et al., 2017; Shi et al., 2019). This persistent OxPhos dominance may reflect metabolic inflexibility that supports bacterial persistence. In parallel, suppression of ion channel and transporter activity-particularly calcium signaling via TRPV4 (Naik et al., 2020; Mitini-Nkhoma et al., 2021)-may compromise phagosome-lysosome fusion and autophagy, essential for intracellular bacterial control. We also observed enrichment of extracellular matrix (ECM) remodeling pathways, consistent with prior reports that excessive collagen deposition shapes granuloma architecture and promotes fibrosis (Marshall et al., 1996; Al Shammari et al., 2015) (Ladel et al., 1995). Together, these alterations suggest that DR-TB is characterized by both metabolic maladaptation and tissue remodeling that reinforce pathogen survival and tissue pathology.

Immunologically, the most striking finding was the depletion of γδ T cells in DR-TB. γδ T cells are pivotal in recognizing Mtb phosphoantigens independently of MHC presentation, producing IFN-γ and TNF-α to activate macrophages, and directly lysing infected cells (D’Souza et al., 1997; Hu et al., 2023). Their reduction in DR-TB aligns with impaired early containment and poorer treatment outcomes, consistent with reports that adoptive transfer of γδ T cells improves responses in MDR-TB patients (Liang et al., 2021). This highlights γδ T cell augmentation as a rational host-directed therapeutic strategy. Among the diagnostic gene signature, *ARHGEF5* strongly linked to immunity through regulation of dendritic cell migration via RhoA activation, crucial for innate immune responses (Wang et al., 2009). PTGES3L may integrate oxidative DNA damage signals with immune gene activation, potentially modulating macrophage phagocytosis and inflammation (de Vries et al., 2021). *GAL3ST1* promotes immune evasion in cancers through HIF1-sulfatide axes and correlates with immune cell infiltration, suggesting anti-infection potential (Díaz-Alvarez and Ortega, 2017). RANBP17 plays a key role in anti-viral defense via nucleocytoplasmic transport (Mandic et al., 2022). *ACTA2-AS1* potentially regulates immune responses in infections, as pathogenic mycoplasmas alter similar lncRNAs for immune modulation and chromatin functions (Ramos et al., 2023). Their inclusion suggests that immune dysregulation in DR-TB extends beyond innate lymphocytes to broader interferon and antigen-presenting pathways, reinforcing the potential of immune-focused biomarkers.

From a translational perspective, the development of an 8-gene BALF-derived signature achieving an AUC of 0.837 represents a promising step toward host-based diagnostics. Unlike conventional genotypic resistance testing, which depends on sputum quality and prior drug exposure, host-response signatures may provide complementary information on treatment-resistant disease. However, external validation in independent cohorts with standardized clinical outcomes will be essential before clinical deployment. Integration with rapid sputum-based assays could further enhance diagnostic performance (Hamada et al., 2022; Parwati et al., 2022; Fonseca et al., 2025).

This study has several limitations. First, the diagnostic classifier was derived computationally from a single-center cohort. Although we implemented a rigorous undersampling strategy to address the class imbalance (49 DR-TB vs. 81 DS-TB) and minimize bias, the lack of an independent external validation cohort remains a major limitation. Consequently, the reported diagnostic performance may reflect cohort-specific features, and the model’s generalizability to populations with different DR-TB prevalence rates warrants further verification in multi-center prospective studies. Second, the cross-sectional design precludes causal inference between microbiome alterations, host responses, and the emergence of drug resistance. Longitudinal sampling before and after treatment initiation would clarify temporal dynamics. Third, while BALF provides a direct window into the airway niche, it may not fully capture systemic immune responses or the gut-lung axis, both of which are increasingly recognized in TB (Alvarado-Peña et al., 2023; Enjeti et al., 2023). Future work should integrate multi-compartmental sampling and functional assays (e.g., γδ T cell functional rescue, phage transduction experiments) to dissect causal mechanisms.

In summary, DR-TB is characterized by synergistic microbial and host alterations, including streptococcal enrichment and phage dysbiosis, metabolic inflexibility with OxPhos dominance, suppression of ion transport pathways, and γδ T cell depletion. Together with an emerging BALF-based host transcriptional signature, these findings provide a foundation for microbiome- and host-directed diagnostics and interventions in tuberculosis drug resistance.

## Data Availability

The datasets presented in this study can be found in online repositories. The names of the repository/repositories and accession number(s) can be found below: https://ngdc.cncb.ac.cn/gsa, PRJCA046985.

## References

[B1] Al Shammari B. Shiomi T. Tezera L. Bielecka M. K. Workman V. Sathyamoorthy T. . (2015). The extracellular matrix regulates granuloma necrosis in tuberculosis. J. Infect. Dis. 212, 463–473. doi: 10.1093/infdis/jiv076, PMID: 25676469 PMC4539912

[B2] Alvarado-Peña N. Galeana-Cadena D. Gómez-García I. A. Mainero X. S. Silva-Herzog E. (2023). The microbiome and the gut-lung axis in tuberculosis: interplay in the course of disease and treatment. Front. Microbiol. 14. doi: 10.3389/fmicb.2023.1237998, PMID: 38029121 PMC10643882

[B3] Azimi T. Mosadegh M. Nasiri M. J. Sabour S. Karimaei S. Nasser A. (2019). Phage therapy as a renewed therapeutic approach to mycobacterial infections: a comprehensive review. Infect. Drug Resist. 12, 2943–2959. doi: 10.2147/idr.S218638, PMID: 31571947 PMC6756577

[B4] Beghini F. McIver L. J. Blanco-Míguez A. Dubois L. Asnicar F. Maharjan S. . (2021). Integrating taxonomic, functional, and strain-level profiling of diverse microbial communities with bioBakery 3. Elife 10, e65088. doi: 10.7554/eLife.65088, PMID: 33944776 PMC8096432

[B5] Bolger A. M. Lohse M. Usadel B. (2014). Trimmomatic: a flexible trimmer for Illumina sequence data. Bioinformatics 30, 2114–2120. doi: 10.1093/bioinformatics/btu170, PMID: 24695404 PMC4103590

[B6] Caspi R. Billington R. Keseler I. M. Kothari A. Krummenacker M. Midford P. E. . (2020). The MetaCyc database of metabolic pathways and enzymes - a 2019 update. Nucleic Acids Res. 48, D445–d453. doi: 10.1093/nar/gkz862, PMID: 31586394 PMC6943030

[B7] Cook G. M. Hards K. Dunn E. Heikal A. Nakatani Y. Greening C. . (2017). Oxidative phosphorylation as a target space for tuberculosis: success, caution, and future directions. Microbiol. Spectr. 5, 1–22. doi: 10.1128/microbiolspec.TBTB2-0014-2016, PMID: 28597820 PMC5480969

[B8] D’Souza C. D. Cooper A. M. Frank A. A. Mazzaccaro R. J. Bloom B. R. Orme I. M. (1997). An anti-inflammatory role for gamma delta T lymphocytes in acquired immunity to Mycobacterium tuberculosis. J. Immunol. 158, 1217–1221. doi: 10.4049/jimmunol.158.3.1217, PMID: 9013962

[B9] Dedrick R. M. Guerrero-Bustamante C. A. Garlena R. A. Russell D. A. Ford K. Harris K. . (2019). Engineered bacteriophages for treatment of a patient with a disseminated drug-resistant Mycobacterium abscessus. Nat. Med. 25, 730–733. doi: 10.1038/s41591-019-0437-z, PMID: 31068712 PMC6557439

[B10] de Vries S. Benes V. Naarmann-de Vries I. S. Rücklé C. Zarnack K. Marx G. . (2021). P23 acts as functional RBP in the macrophage inflammation response. Front. Mol. Biosci. 8, 2021. doi: 10.3389/fmolb.2021.625608, PMID: 34179071 PMC8226254

[B11] Diao Z. Lai H. Han D. Yang B. Zhang R. Li J. (2023). Validation of a metagenomic next-generation sequencing assay for lower respiratory pathogen detection. Microbiol. Spectr. 11, e0381222. doi: 10.1128/spectrum.03812-22, PMID: 36507666 PMC9927246

[B12] Díaz-Alvarez L. Ortega E. (2017). The many roles of galectin-3, a multifaceted molecule, in innate immune responses against pathogens. Mediators Inflammation 2017, 9247574. doi: 10.1155/2017/9247574, PMID: 28607536 PMC5457773

[B13] Du W. Zhao Y. Zhang C. Zhang L. Zhou L. Sun Z. . (2024). Association of bacteriomes with drug susceptibility in lesions of pulmonary tuberculosis patients. Heliyon 10, e37583. doi: 10.1016/j.heliyon.2024.e37583, PMID: 39309911 PMC11414563

[B14] Enjeti A. Sathkumara H. D. Kupz A. (2023). Impact of the gut-lung axis on tuberculosis susceptibility and progression. Front. Microbiol. 14. doi: 10.3389/fmicb.2023.1209932, PMID: 37485512 PMC10358729

[B15] Fonseca K. L. Lozano J. J. Despuig A. Habgood-Coote D. Sidorova J. Aznar D. . (2025). Unravelling the transcriptome of the human tuberculosis lesion and its clinical implications. Nat. Commun. 16, 5028. doi: 10.1038/s41467-025-60255-w, PMID: 40447570 PMC12125219

[B16] Gillespie M. Jassal B. Stephan R. Milacic M. Rothfels K. Senff-Ribeiro A. . (2022). The reactome pathway knowledgebase 2022. Nucleic Acids Res. 50, D687–d692. doi: 10.1093/nar/gkab1028, PMID: 34788843 PMC8689983

[B17] Gupta N. Kumar R. Agrawal B. (2018). New players in immunity to tuberculosis: the host microbiome, lung epithelium, and innate immune cells. Front. Immunol. 9. doi: 10.3389/fimmu.2018.00709, PMID: 29692778 PMC5902499

[B18] Haddock N. L. Barkal L. J. Ram-Mohan N. Kaber G. Chiu C. Y. Bhatt A. S. . (2023). Phage diversity in cell-free DNA identifies bacterial pathogens in human sepsis cases. Nat. Microbiol. 8, 1495–1507. doi: 10.1038/s41564-023-01406-x, PMID: 37308590 PMC10911932

[B19] Hamada Y. Penn-Nicholson A. Krishnan S. Cirillo D. M. Matteelli A. Wyss R. . (2022). Are mRNA based transcriptomic signatures ready for diagnosing tuberculosis in the clinic? - A review of evidence and the technological landscape. EBioMedicine 82, 104174. doi: 10.1016/j.ebiom.2022.104174, PMID: 35850011 PMC9294474

[B20] Han D. Liu C. Yang B. Yu F. Liu H. Lou B. . (2025). Metagenomic fingerprints in bronchoalveolar lavage differentiate pulmonary diseases. NPJ Digit Med. 8, 599. doi: 10.1038/s41746-025-01977-5, PMID: 41057624 PMC12504733

[B21] Ho S. F. S. Wheeler N. E. Millard A. D. van Schaik W. (2023). Gauge your phage: benchmarking of bacteriophage identification tools in metagenomic sequencing data. Microbiome 11, 84. doi: 10.1186/s40168-023-01533-x, PMID: 37085924 PMC10120246

[B22] Hong B. Y. Maulén N. P. Adami A. J. Granados H. Balcells M. E. Cervantes J. (2016). Microbiome changes during tuberculosis and antituberculous therapy. Clin. Microbiol. Rev. 29, 915–926. doi: 10.1128/cmr.00096-15, PMID: 27608937 PMC5010754

[B23] Howard N. C. Khader S. A. (2020). Immunometabolism during Mycobacterium tuberculosis Infection. Trends Microbiol. 28, 832–850. doi: 10.1016/j.tim.2020.04.010, PMID: 32409147 PMC7494650

[B24] Hu Y. Hu Q. Li Y. Lu L. Xiang Z. Yin Z. . (2023). γδ T cells: origin and fate, subsets, diseases and immunotherapy. Signal Transduction Targeted Ther. 8, 434. doi: 10.1038/s41392-023-01653-8, PMID: 37989744 PMC10663641

[B25] Kanehisa M. Furumichi M. Tanabe M. Sato Y. Morishima K. (2017). KEGG: new perspectives on genomes, pathways, diseases and drugs. Nucleic Acids Res. 45, D353–d361. doi: 10.1093/nar/gkw1092, PMID: 27899662 PMC5210567

[B26] Kim D. Paggi J. M. Park C. Bennett C. Salzberg S. L. (2019). Graph-based genome alignment and genotyping with HISAT2 and HISAT-genotype. Nat. Biotechnol. 37, 907–915. doi: 10.1038/s41587-019-0201-4, PMID: 31375807 PMC7605509

[B27] Ladel C. H. Blum C. Dreher A. Reifenberg K. Kaufmann S. H. (1995). Protective role of gamma/delta T cells and alpha/beta T cells in tuberculosis. Eur. J. Immunol. 25, 2877–2881. doi: 10.1002/eji.1830251025, PMID: 7589086

[B28] Lange C. Dheda K. Chesov D. Mandalakas A. M. Udwadia Z. Horsburgh C. R. Jr. (2019). Management of drug-resistant tuberculosis. Lancet 394, 953–966. doi: 10.1016/s0140-6736(19)31882-3, PMID: 31526739 PMC11524526

[B29] Liang J. Fu L. Li M. Chen Y. Wang Y. Lin Y. . (2021). Allogeneic Vγ9Vδ2 T-cell therapy promotes pulmonary lesion repair: an open-label, single-arm pilot study in patients with multidrug-resistant tuberculosis. Front. Immunol. 12. doi: 10.3389/fimmu.2021.756495, PMID: 34975844 PMC8715986

[B30] Liao Y. Smyth G. K. Shi W. (2014). featureCounts: an efficient general purpose program for assigning sequence reads to genomic features. Bioinformatics 30, 923–930. doi: 10.1093/bioinformatics/btt656, PMID: 24227677

[B31] Lin Z. Jiang Y. Liu H. Yang J. Yang B. Zhang K. . (2025). Airway microbiota and immunity associated with chronic obstructive pulmonary disease severity. J. Trans. Med. 23, 962. doi: 10.1186/s12967-025-06986-2, PMID: 40859318 PMC12382185

[B32] Lin D. Wang X. Li Y. Wang W. Li Y. Yu X. . (2021). Sputum microbiota as a potential diagnostic marker for multidrug-resistant tuberculosis. Int. J. Med. Sci. 18, 1935–1945. doi: 10.7150/ijms.53492, PMID: 33850462 PMC8040397

[B33] Love M. I. Huber W. Anders S. (2014). Moderated estimation of fold change and dispersion for RNA-seq data with DESeq2. Genome Biol. 15, 550. doi: 10.1186/s13059-014-0550-8, PMID: 25516281 PMC4302049

[B34] Mandic R. Marquardt A. Terhorst P. Ali U. Nowak-Rossmann A. Cai C. . (2022). The importin beta superfamily member RanBP17 exhibits a role in cell proliferation and is associated with improved survival of patients with HPV+ HNSCC. BMC Cancer 22, 785. doi: 10.1186/s12885-022-09854-0, PMID: 35850701 PMC9290296

[B35] Marshall B. G. Wangoo A. Cook H. T. Shaw R. J. (1996). Increased inflammatory cytokines and new collagen formation in cutaneous tuberculosis and sarcoidosis. Thorax 51, 1253–1261. doi: 10.1136/thx.51.12.1253, PMID: 8994525 PMC472773

[B36] Mick E. Tsitsiklis A. Kamm J. Kalantar K. L. Caldera S. Lyden A. . (2023). Integrated host/microbe metagenomics enables accurate lower respiratory tract infection diagnosis in critically ill children. J. Clin. Invest. 133, e165904. doi: 10.1172/jci165904, PMID: 37009900 PMC10065066

[B37] Miller S. Naccache S. N. Samayoa E. Messacar K. Arevalo S. Federman S. . (2019). Laboratory validation of a clinical metagenomic sequencing assay for pathogen detection in cerebrospinal fluid. Genome Res. 29, 831–842. doi: 10.1101/gr.238170.118, PMID: 30992304 PMC6499319

[B38] Mitini-Nkhoma S. C. Chimbayo E. T. Mzinza D. T. Mhango D. V. Chirambo A. P. Mandalasi C. . (2021). Something old, something new: ion channel blockers as potential anti-tuberculosis agents. Front. Immunol. 12. doi: 10.3389/fimmu.2021.665785, PMID: 34248944 PMC8264357

[B39] Naik S. K. Pattanaik K. Eich J. Sparr V. Hauptmann M. Kalsdorf B. . (2020). Differential roles of the calcium ion channel TRPV4 in host responses to mycobacterium tuberculosis early and late in infection. iScience 23. doi: 10.1016/j.isci.2020.101206, PMID: 32535021 PMC7300151

[B40] Parwati I. Pitaloka D. A. E. Chaidir L. (2022). Transcriptional biomarkers for treatment monitoring of pulmonary drug-resistant tuberculosis: protocol for a prospective observational study in Indonesia. Trop. Med. Infect. Dis. 7, 326. doi: 10.3390/tropicalmed7110326, PMID: 36355869 PMC9698426

[B41] Ramos E. I. Veerapandian R. Das K. Chacon J. A. Gadad S. S. Dhandayuthapani S. (2023). Pathogenic mycoplasmas of humans regulate the long noncoding RNAs in epithelial cells. Non-coding RNA Res. 8, 282–293. doi: 10.1016/j.ncrna.2023.03.002, PMID: 36970372 PMC10031284

[B42] Ren L. Wang Y. Zhong J. Li X. Xiao Y. Li J. . (2021). Dynamics of the upper respiratory tract microbiota and its association with mortality in COVID-19. Am. J. Respir. Crit. Care Med. 204, 1379–1390. doi: 10.1164/rccm.202103-0814OC, PMID: 34534435 PMC8865718

[B43] Segata N. Izard J. Waldron L. Gevers D. Miropolsky L. Garrett W. S. . (2011). Metagenomic biomarker discovery and explanation. Genome Biol. 12, R60. doi: 10.1186/gb-2011-12-6-r60, PMID: 21702898 PMC3218848

[B44] Shahzad M. Saeed M. Amin H. Binmadi N. Ullah Z. Bibi S. . (2024). The oral microbiome of newly diagnosed tuberculosis patients; a pilot study. Genomics 116, 110816. doi: 10.1016/j.ygeno.2024.110816, PMID: 38431030

[B45] Shi L. Jiang Q. Bushkin Y. Subbian S. Tyagi S. (2019). Biphasic Dynamics of Macrophage Immunometabolism during Mycobacterium tuberculosis Infection. mBio 10, 1–19. doi: 10.1128/mBio.02550-18, PMID: 30914513 PMC6437057

[B46] Steen C. B. Liu C. L. Alizadeh A. A. Newman A. M. (2020). Profiling cell type abundance and expression in bulk tissues with CIBERSORTx. Methods Mol. Biol. 2117, 135–157. doi: 10.1007/978-1-0716-0301-7_7, PMID: 31960376 PMC7695353

[B47] Subramanian A. Tamayo P. Mootha V. K. Mukherjee S. Ebert B. L. Gillette M. A. . (2005). Gene set enrichment analysis: a knowledge-based approach for interpreting genome-wide expression profiles. Proc. Natl. Acad. Sci. U.S.A. 102, 15545–15550. doi: 10.1073/pnas.0506580102, PMID: 16199517 PMC1239896

[B48] Sun Q. Teng R. Shi Q. Liu Y. Cai X. Yang B. . (2025). Clinical implement of Probe-Capture Metagenomics in sepsis patients: A multicentre and prospective study. Clin. Transl. Med. 15, e70297. doi: 10.1002/ctm2.70297, PMID: 40181528 PMC11968419

[B49] Truong D. T. Franzosa E. A. Tickle T. L. Scholz M. Weingart G. Pasolli E. . (2015). MetaPhlAn2 for enhanced metagenomic taxonomic profiling. Nat. Methods 12, 902–903. doi: 10.1038/nmeth.3589, PMID: 26418763

[B50] Valdez-Palomares F. Muñoz Torrico M. Palacios-González B. Soberón X. Silva-Herzog E. (2021). Altered microbial composition of drug-sensitive and drug-resistant TB patients compared with healthy volunteers. Microorganisms 9, 1762. doi: 10.3390/microorganisms9081762, PMID: 34442841 PMC8398572

[B51] Walker T. M. Miotto P. Köser C. U. Fowler P. W. Knaggs J. Iqbal Z. . (2022). The 2021 WHO catalogue of Mycobacterium tuberculosis complex mutations associated with drug resistance: A genotypic analysis. Lancet Microbe 3, e265–e273. doi: 10.1016/s2666-5247(21)00301-3, PMID: 35373160 PMC7612554

[B52] Wang Z. Kumamoto Y. Wang P. Gan X. Lehmann D. Smrcka A. V. . (2009). Regulation of immature dendritic cell migration by rhoA guanine nucleotide exchange factor arhgef5*. J. Biol. Chem. 284, 28599–28606. doi: 10.1074/jbc.M109.047282, PMID: 19713215 PMC2781403

[B53] Wu J. Liu W. He L. Huang F. Chen J. Cui P. . (2013). Sputum microbiota associated with new, recurrent and treatment failure tuberculosis. PloS One 8, e83445. doi: 10.1371/journal.pone.0083445, PMID: 24349510 PMC3862690

[B54] Yang F. Labani-Motlagh A. Bohorquez J. A. Moreira J. D. Ansari D. Patel S. . (2024a). Bacteriophage therapy for the treatment of Mycobacterium tuberculosis infections in humanized mice. Commun. Biol. 7, 294. doi: 10.1038/s42003-024-06006-x, PMID: 38461214 PMC10924958

[B55] Yang J. Li J. Zhang L. Shen Z. Xiao Y. Zhang G. . (2024b). Highly diverse sputum microbiota correlates with the disease severity in patients with community-acquired pneumonia: a longitudinal cohort study. Respir. Res. 25, 223. doi: 10.1186/s12931-024-02821-2, PMID: 38811936 PMC11137881

[B56] You H. Yang B. Liu H. Wu W. Yu F. Lin N. . (2025). Unravelling distinct patterns of metagenomic surveillance and respiratory microbiota between two P1 genotypes of Mycoplasma pneumoniae. Emerg. Microbes Infect. 14, 2449087. doi: 10.1080/22221751.2024.2449087, PMID: 39760260 PMC11730683

[B57] Zhang S. Chen X. Jin E. Wang A. Chen T. Zhang X. . (2025). The GSA family in 2025: A broadened sharing platform for multi-omics and multimodal data. Genomics Proteomics Bioinf. 23, 1–8. doi: 10.1093/gpbjnl/qzaf072, PMID: 40857552 PMC12451262

[B58] Zou X. Cao H. Hong L. Suo L. Wang C. Chang K. . (2025a). Enrichment of Streptococcus oralis in respiratory microbiome enhance innate immunity and protects against influenza infection. Signal Transduct Target Ther. 10, 272. doi: 10.1038/s41392-025-02365-x, PMID: 40858544 PMC12381301

[B59] Zou X. Yan M. Wang Y. Ni Y. Zhao J. Lu B. . (2025b). Accurate diagnosis of lower respiratory infections using host response and respiratory microbiome from a single metatranscriptome test of bronchoalveolar lavage fluid. Adv. Sci. (Weinh) 12, e2405087. doi: 10.1002/advs.202405087, PMID: 39692191 PMC11809327

