# Correction: Metagenomic and metatranscriptomic profiling of bronchoalveolar lavage fluid identifies microbial and host biomarkers of drug-resistant tuberculosis

**DOI:** 10.3389/fcimb.2026.1826950

**Published:** 2026-03-26

**Authors:** Haiqing Zhang, Limao Zhang, Bin Yang, Chunjing Gao, Huimei Liu, Yunshi Zhang, Xiaoyou Chen

**Affiliations:** 1 Department of Tuberculosis, Xuzhou Hospital, Beijing Ditan Hospital Affiliated to Capital Medical University, Xuzhou, Jiangsu, China; 2 Center for Infectious Diseases, Vision Medicals Co., Ltd, Guangzhou, Guangdong, China; 3 Department of Science and Education, Xuzhou Hospital, Beijing Ditan Hospital Affiliated to Capital Medical University, Xuzhou, Jiangsu, China; 4 Xuzhou Hospital, Beijing Ditan Hospital Affiliated to Capital Medical University, Xuzhou, Jiangsu, China

**Keywords:** drug resistance, host response, microbiome, *Mycobacterium tuberculosis*, transcriptomics

Affiliation “Xuzhou Hospital, Beijing Ditan Hospital Affiliated to Capital Medical University, Xuzhou, Jiangsu, China” was erroneously given as “Infectious Diseases Department, Beijing Ditan Hospital, Capital Medical University, Beijing, China” and “Xuzhou Hospital for Infectious Diseases, Xuzhou, Jiangsu, China”.

The original version of this article has been updated.

