# Supplementary material for: Metagenomic and metatranscriptomic profiling of bronchoalveolar lavage fluid identifies microbial and host biomarkers of drug-resistant tuberculosis
Source: Front Cell Infect Microbiol. 2026 Jan 29;15:1726935. doi: 10.3389/fcimb.2025.1726935 (PMC12894345; doi:10.3389/fcimb.2025.1726935)
Supplement: Supplementary file 1 [file DataSheet1.docx]

Supplementary Material

# Supplementary Figures


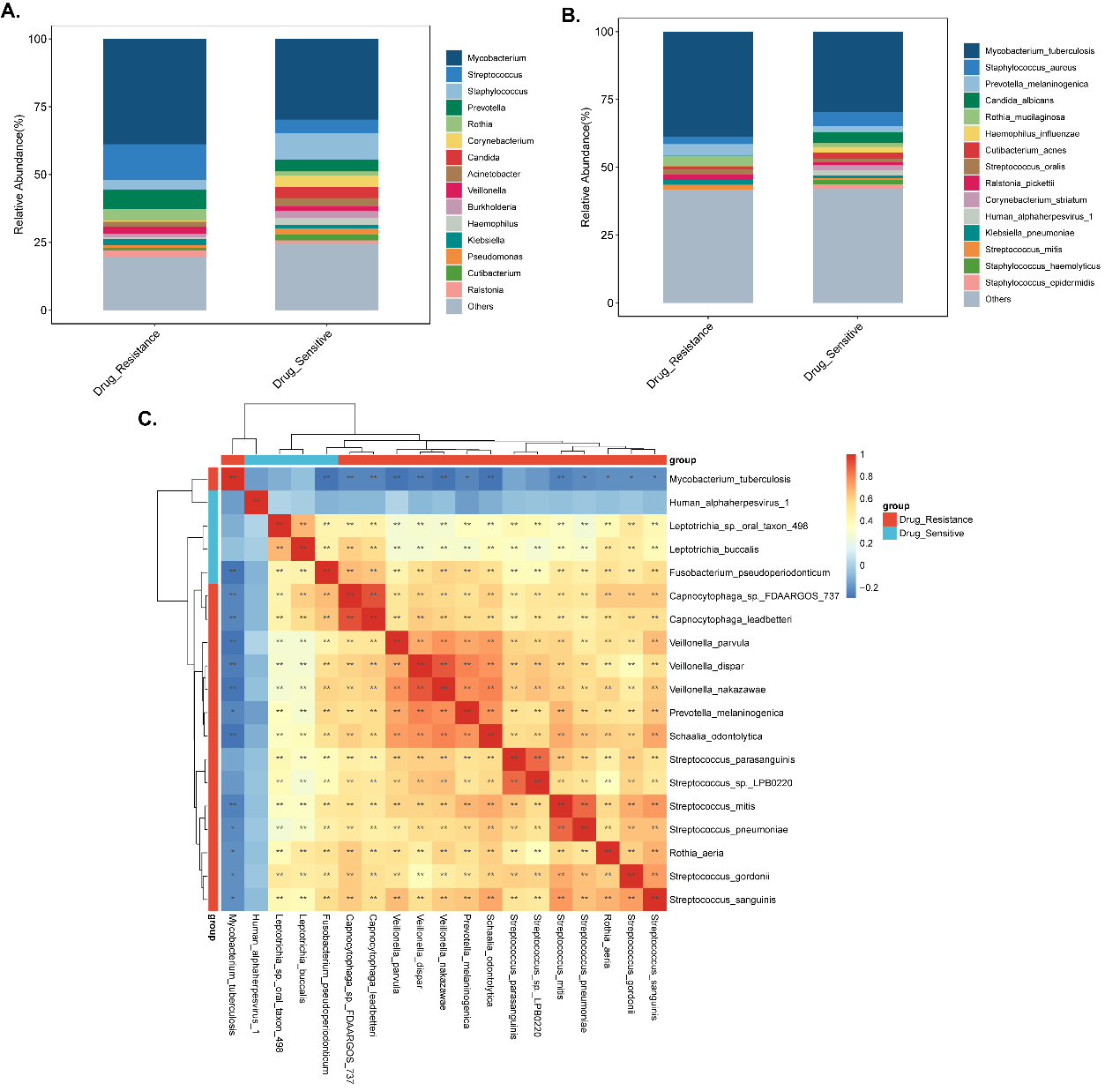


**Supplementary Figure 1.** Comparison of respiratory microbial composition among two groups. (A-B) Relative abundance of different microbial genus (A) and species (B) in two groups. (C) Heatmap demonstrating the correlations between microbial species that showed significantly different expressions in two groups.


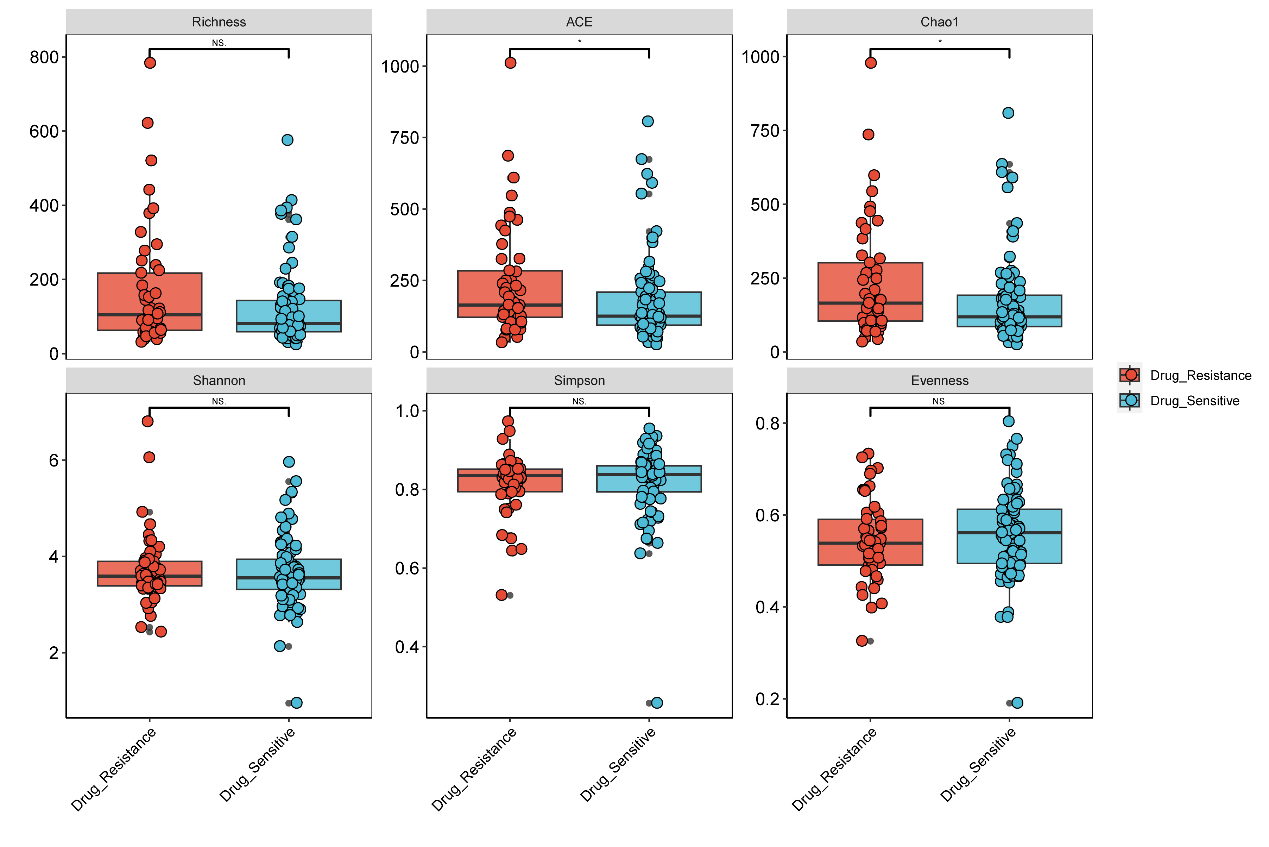


**Supplementary Figure 2.** Comparison of respiratory bacteriophages composition among DR-TB and DS-TB. alpha-diversity was evaluated by richness, ACE, Chao1, Shannon, Simpson, and evenness indexes in BALF samples from DR-TB and DS-TB, respectively. Drug-resistant samples are denoted in red; drug-sensitive samples in blue. “*” Represents significance between two groups (*: p-value < 0.05) and NS. represents no significance between two groups. DR-TB, drug-resistant tuberculosis; DS-TB, drug-sensitive tuberculosis.


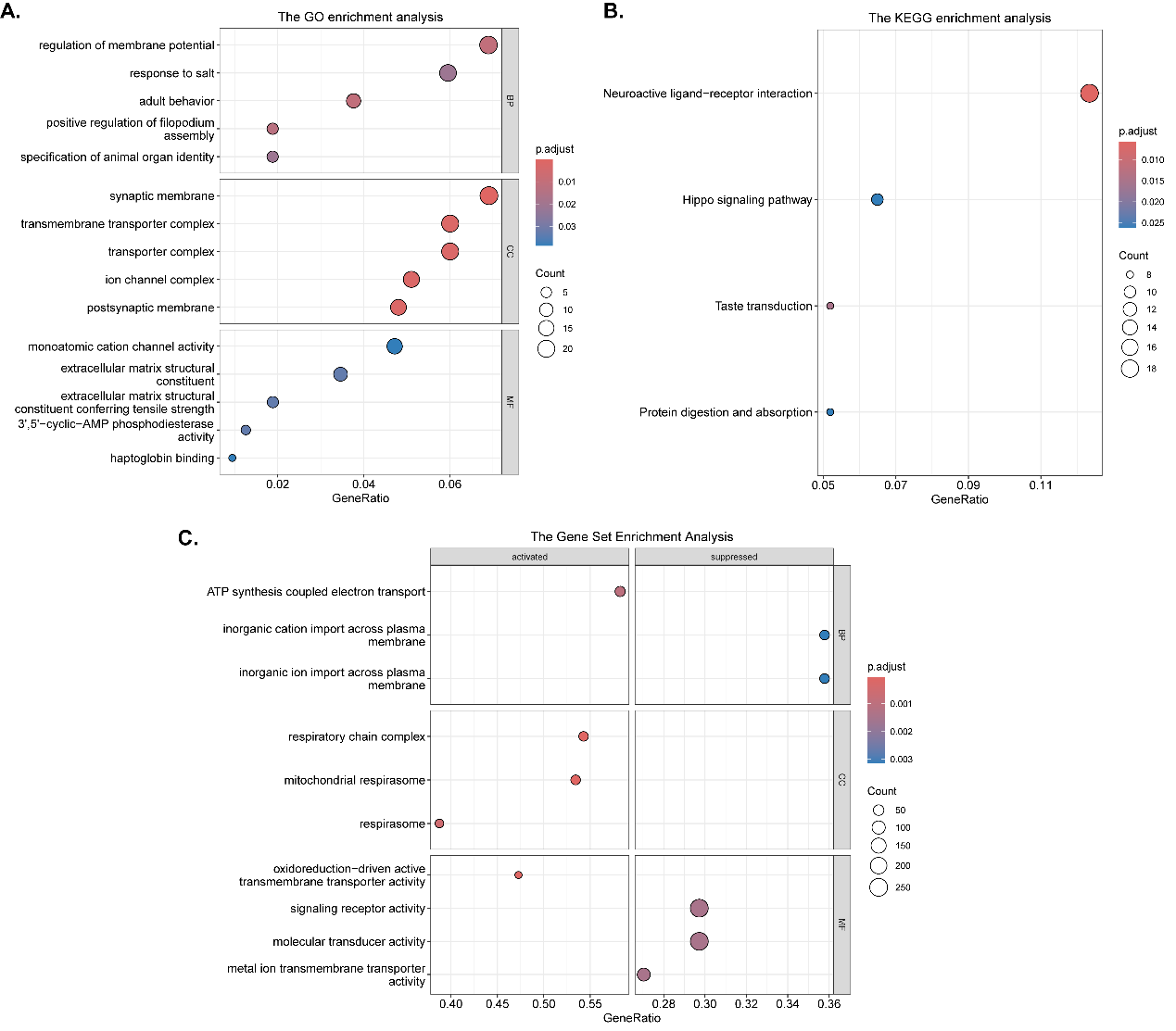


**Supplementary Figure 3.** Gene functional classification of discrepantly expressed genes among two groups using transcriptomics. (A-B) Gene ontology enrichment (A) and KEGG (B) enrichment of discrepantly expressed genes. (C) GO enrichment of Gene Set Enrichment Analysis between DT-TB versus DS-TB. DR-TB, drug-resistant tuberculosis; DS-TB, drug-sensitive tuberculosis.
